# Potential bioremediation of lead and phenol by sunflower seed husk and rice straw-based biochar hybridized with bacterial consortium: a kinetic study

**DOI:** 10.1038/s41598-023-49036-x

**Published:** 2023-12-11

**Authors:** Eman H. El-Gamal, Mohamed Rashad, Maher E. Saleh, Sahar Zaki, Marwa Eltarahony

**Affiliations:** 1 Land and Water Technologies Department, Arid Lands Cultivation Research Institute (ALCRI), City of Scientific Research and Technological Applications (SRTA-City), New Borg El-Arab City, 21934 Alexandria Egypt; 2 Department of Soils and Water Sciences, Faculty of Agriculture, Alexandria University, El-Shatby, 21545 Alexandria Egypt; 3 Environmental Biotechnology Department, Genetic Engineering and Biotechnology Research Institute (GEBRI), City of Scientific Research and Technological Applications (SRTA-City), New Borg El-Arab City, 21934 Alexandria Egypt

**Keywords:** Biomineralization, Environmental sciences

## Abstract

Environmental pollution is a global phenomenon and troublesome fact that poses a grave risk to all living entities. Via coupling carbonaceous feedstocks with outstanding microbial activity, kinetic experiments were established using the consortium of *Proteus mirabilis* and *Raoultella planticola*, biochar-derived sunflower seed husk (SHB) and rice straw (RSB), and their composites, which investigated at 30 °C (150 rpm) to eliminate 700 mg L^−1^ lead (120 h) and phenol (168 h) from synthetic wastewater. The derived biochars physicochemical properties of were studied. According to adsorption capacity (q_*e*_), consortium-SHB composites and consortium-RSB composites removed lead completely (70 mg g^−1^) within 48 h and 66 h, respectively. Besides, phenol was remediated entirely after 42 h and 48 h by both composite systems (69.90 mg g^−1^), respectively, comparing with bacterial consortium only or parent SHB and RSB. Moreover, four kinetic models were studied to describe the bioremediation process. Fractional power and Elovich models could be recommended for describing the adsorption kinetics for lead and phenol removal by the studied biomaterials with high correlation coefficient (R^2^ ≥ 0.91 for Pb^2+^ and ≥ 0.93 for phenol) and lower residual root mean square error (RMSE) and chi-square (*X*^2^). Overall, bacterial consortium-biochar composites exhibited greater remediation of lead and phenol than the sum of each single bacterial consortium and biochar systems; reflecting synergistic interaction of adsorptive capability of biochar and metabolic performance of bacterial consortium, as denoted by scanning electron microscopy (SEM) and energy-dispersive X-ray spectroscopy (EDX). The current study addressed the successful design of employing functional remediating consortium immobilized on waste biomass-derived biochar as a conducive alternative eco-sorbent and economic platform to detoxify organic and inorganic pollutants.

## Introduction

Wastewater contains various toxins derived from human industrial activities that must be remediated to face water scarcity. Organic and inorganic pollutants in wastewater demand special consideration to eliminate their toxicity and zeta potential (ζ-potential) for bioaccumulation along the food chain, which harms all living organisms^1^. Heavy metals are introduced into the environment from point and non-point sources as discharges from different industrial sectors like metal extraction, metal plating processes, battery manufacturing, steel pipes, and erosion of natural deposits. Lead (Pb) is classified among the most toxic and carcinogenic heavy metals along with mercury, nickel, arsenic, cadmium, cobalt, chromium, and aluminum, which could cause severe ecological and health problems^2–5^. Moreover, organic pollutants are generated from civilization, industrial effluents, and agricultural practices, such as biphenyls, pesticides, fertilizers, oils, greases, hydrocarbons, plasticizers, detergents, and medicines. Among them are phenolic compounds (e.g., phenol), which are a building block for several pharmaceuticals, disinfectants, and resin products^6^. The major threat of both heavy metals and phenolic compounds lies behind their persistence in the environment and tolerance for solar or microbial decomposition, which leads to accumulation gradually in cellular compartments of aquatic creatures and eventually reaches human beings through the food web^7^. Hereby, the US Environmental Protection Agency (EPA) and World Health Organization (WHO) classified both Pb and phenol as high-priority pollutants and reported their limits in the environment as 15 and 0.1 ppm, respectively^8^.

Therefore, several treatment approaches have been developed to purify wastewater from such a burden, particularly with the growing population and scarcity of water sources. Multiple conventional physicochemical techniques have been used for toxic compounds removal from wastewater, including adsorption onto different matrices, precipitation, ion exchange, chemical oxidation, solvent extraction or irradiation, coagulation, column filtration, and membrane filtration^3,4,9,10^. However, biological means, either micro-remediation or phytoremediation, frequently proved their higher efficacy. While phytoremediation has drawbacks including long remediation times and susceptibility to seasonal changes, micro-remediation tackles such issues. Bioremediation via microorganisms is considered one of the cheapest possible solutions to resolve pollution problems through numerous versatile metabolism-dependent and metabolism-independent processes such as bioconversion, bio-precipitation, bioaccumulation, and biosorption^7,11^. Nonetheless, recent studies have been focused on developing advanced methods to ameliorate microbial efficiency in waste remediation processes. Unfortunately, some of these methods exhibited a number of disadvantages, including the loss or reduction of microbial activity, and the challenge of separation and recovery after treatment^10^_._ As a result, immobilization techniques are introduced to address these shortcomings, which increase effectiveness and reproducibility through maintaining microbial activity and protecting cells from the danger of the surrounding environment.

Numerous materials have been extensively used as carriers for immobilizing microorganisms, including polyvinyl alcohol, chitosan, alginate, gelatin, agar, and porous ceramics. Several advantages and disadvantages of these materials have been reported related to biodegradable resistance, cost, fixed difficulty, and mechanical strength^10,12^ Recently, carbon-based materials have attracted great attention, especially those derived from agricultural wastes such as biochar. Biochar is a solid by-product material produced by the pyrolysis process of organic waste in an oxygen-limited environment^13–15^. It is a black carbon-rich substance with unique properties for various applications, such as high cation exchange capacity (CEC), large surface area, high porosity, functional groups, and stability^14,16^. Such properties can be influenced by the type of biomass used, the pyrolysis formulation (temperature, heating rate, and residence time), and the modification technique (chemical, physical, or biological). Many scientists have reported the high adsorption efficiency of biochar in the removal of various contaminants^17^. Numerous adsorption mechanisms depend on biochar surface characteristics and pollutant physicochemical properties, including electrostatic interaction, ion exchange, pore filling, and precipitation^1,17,18^. It is recognized that the biochar adsorption capacities for pollutants vary significantly depending on biochar properties, pollutant characteristics, and the competition between pollutants on the biochar surface^17^. Therefore, it is plausible to describe biochar as an eco-friendly cheap multipurpose material for climate change alleviation, recovering soil efficiency, and managing toxic metals and organic pollutants, which eventually could be employed as a green alternative to costly conventional sorbents like activated carbon^1,14,16^.

Based on the preceding background, linking biochar characteristic features with microbes to enhance the remediation capacity through immobilization is considered a promising approach for eliminating various contaminants from wastewater. Via such composites, the microbial number, microbial metabolic capability, reusability, and reproducibility could be enhanced, compared to sole-material-dependent technologies. Hence, the present study focused on assessing the kinetic analysis of bacterial consortium-biochar composites in the remediation process. It commenced with preparing and characterizing two biochar types (sunflower seed husk “SH” and rice straw “RS”). Followed by formulating of bacterial consortium-biochar composites by immobilizing *Proteus mirabilis* (10B) and *Raoultella planticola* (VIP) strains on both biochar types and examining their bioremediation capacity in the removal of lead and phenol as models of inorganic and organic pollutants, respectively. In parallel, the remediation capacity of the bacterial consortium was also compared with both biochar types. Finally, Fractional Power, Elovich, Pseudo-first-order kinetic and, Pseudo-second-order kinetic analysis were studied.

## Materials and methods

### Biochar production and characterization

Two agricultural residues from sunflower seed husk (SH) and rice straw (RS) were used to prepare biochar. The residues were washed with tap water followed by deionized water to eliminate undesirable materials. Then the oven dried materials (at 80 °C for 48 h) were employed to produce biochar using muffle furnace at 600 ± 5 °C with a residence time of 30 min under a partial condition of oxygen^19^. The yielded biochars were cooled under environmental lab conditions (25 °C). The biochar samples were ground, passed through a 0.5 mm sieve, and stored in plastic bags for further uses. The derived biochars were entitled SHB and RSB for sunflower seed husk and rice straw biochar, respectively. The basic parameters of the obtained biochars (e.g., ash content, pH values, electric conductivity “EC”, cation exchange capacity “CEC”, surface area parameters “EBT”, and zeta potential “ζ-potential”) were examined. The ash content of both biochars (SHB and RSB) was determined by dry incineration at 600 °C for 12 h in the air using a muffle furnace. The ash percentage was calculated as follows:^15^

1 $$Ash \left(\mathrm{\%}\right)=\left(\frac{{W}_{f }}{{W}_{i}}\right)*100$$

where W_*i*_ and W_*f*_ are the initial and final weight of materials before and after burning (g), respectively.

To determine biochar pH and EC, one percent of biochar-water suspension (w/v) was agitated for 20 min at 90 °C and the pH was measured using a Professional Multi-Parameter (Bench Meter with GLP—AD8000 model)^15^. Cation exchange capacity of both biochars was measured as defined by Gaskin et al.^20^ using the method of a modified ammonium‐acetate compulsory (CEC-NH_4_) displacement method. After the biochar surface was saturated with 1M sodium acetate (pH 8.2), the biochar samples were washed with ethanol to eliminate the excess sodium ions from the biochar surface. Following that, the sodium ions were moved by a neutral ammonium-acetate solution (1 M), and the released amount of sodium ions was measured by atomic absorption spectroscopy (AAS—ZEENIT 700—Analytik Jena).

The surface area of the two biochars type and their pore diameter and pore volume were determined by Brunauer–Emmett–Teller (BET) method equation using the N_2_ adsorption–desorption isotherm at 77 K on a gas sorption analyzer (Micro-trac MRB BELSORP-mini X, Japan).

The functional groups of biochar were identified by the Fourier transform infrared (FT-IR) spectrum technique using a Shimadzu FT/IR-5300, Tokyo, Japan. The spectral range under investigation was 4000–400 cm^−1^ at room temperature. A dry potassium bromide (KBr) disk method was followed by mixing small amounts of biochar samples, which were subsequently compressed into a disk that was less than 1 mm thick.

Biochar suspension (0.1 g: 200 water, 0.05% suspension) was prepared to measure ζ-potential after shaking at 150 rpm for 12 h using Zetasizer (ZP-Malvern Panalytical-UK) according to Hong et al.^21^.

The surface morphology of gold-coated samples was investigated by scanning electron microscopy (SEM) and energy-dispersive X-ray spectroscopy (EDX) to assess the mineral content using a JEOL, Model JSM–IT200, Japan. The images were taken before and after the lead and phenol sorption prosses at various magnification scales using photographic techniques at 20.0 kV.

### Bacterial cultures

The bacterial consortium, consisting of *Proteus mirabilis* (10B) and *Raoultella planticola* (VIP) strains, were formerly identified and submitted in the GenBank under accession numbers of KY964505 and MK551748, respectively. Both strains revealed renowned physiological properties and were employed previously in different bioremediation studies^8,22^. The strains were cultivated for 24 h at 30 °C, 150 rpm on nutrient broth with the following composition: beef extract (1 g L^−1^), yeast extract (2 g L^−1^), peptone (5 g L^−1^) and NaCl (5 g L^−1^) at pH = 7. After incubation, cells were collected by centrifugation at 200 rpm for 10 min., then washed with 0.85% sterile normal saline and suspended in the same ratios to set a final bacterial concentration of approximately 2 × 10^9^ CFU/ml.

### Bacterial consortium immobilized on biochar

For immobilization, mixed bacterial consortium (OD600 ≈ 1) was loaded by physical adsorption with biochar as follows, 10 g of each biochar was mixed with 100 ml bacterial suspension in a shaking incubator (150 rpm) under 30 °C for 24 h. Then, the biochar immobilized bacterial consortium was filtered, rinsed with sterilized water and then suspended in 0.85% sterile normal saline.

### Lead and phenol removal batch experiment

The batch experiments of lead acetate (PbC_4_H_6_O_4_) or phenol (C_6_H_5_OH) were performed in 100 ml solution of 700 mg L^−1^ (Pb^2+^ or phenol) initial concentration. The experiments were established in four set flasks, namely, the first control set contained Pb/phenol without biochar or bacterial inoculm, the second set contained Pb/phenol inoculated with 10% bacterial consortium, the third set contained biochar (SHB or RSB) only without bacterial inoculum and fourth set contained bacterial consortium-biochar composites (SHB or RSB). The suspension ratio of sorbents to solution was 1: 100 and all sets were adjusted to initial pH = 7 incubated at 30 °C (150 rpm) for 168 and 120 h for lead and phenol, respectively. At each time interval, the suspensions were filtered through a 0.22-μm filter membrane. The residual Pb^2+^ or phenol concentrations were analyzed in the filtrate supernatants using the inductively coupled plasma mass spectrometry (ICP-MS, Agilent ICP-OES 5110DVD) for Pb, while phenol was estimated according to the method previously defined by Der Yang and Humphrey^23^ based on condensation with 4-amino antipyrine followed by oxidation with potassium ferricyanide and the absorbency deliver at 520 nm. To confirm and characterize the removal efficiency of different sorbents in each set, at the end of incubation period, the bioremediated pellets in all examined sets were centrifuged at 12,000 rpm for 20 min, washed, dried, and analyzed by SEM and additionally EDX for lead. All trials were conducted in triplicates and the data was expressed as mean ± standard error of mean. The removal efficiency (R, %) and adsorption capacities at time t (q_t_, mg g^−1^) and at equilibrium (q_*e*_, mg g^−1^) were calculated as follows:

2 $$RE\%=\frac{{C}_{i}-{C}_{f}}{{C}_{i}} \times 100$$

3 $${q}_{t}=\frac{V({C}_{i}-{C}_{f})}{W}$$

4 $${q}_{e}=\frac{V({C}_{i}-{C}_{f})}{W}$$

where C_*i*_ and C_*f*_ were the adsorbate concentration before and after adsorption (mg L^−1^), respectively. V was the volume of the adsorbate solution (L) and W was the adsorbent mass (g).

### Kinetic study

Kinetics of lead and phenol adsorption were performed by applying Fractional Power, Elovic, Pseudo-frist-order and Pseudo-second-order kinetic models to investigate the adsorption process of both pollutants on to different biochar types.

Fractional Power kinetic model:

5 $$ln{ \,q}_{t}= ln\, a + b\, ln t$$

Elovich kinetic model:

6 $${q}_{t}= \beta\, ln\,\left(\alpha \beta \right)+\beta\, lnt$$

Pseudo-first-order kinetic model:

7 $$ln\left({q}_{e}-{q}_{t}\right)= ln{q}_{e} -{ k}_{1}t$$

Pseudo-second-order kinetic model:

8 $$\frac{t}{q}_{t}= \frac{1}{{k}_{2}{q}_{e}^{2}}+\frac{1}{{q}_{e}t}$$

where q_*t*_ and q_*e*_ (mg g^−1^) are the sorption capacity of both adsorbed on adsorbent materials at time t and at equilibrium, respectively; a (mg g^−1^) and b (h^−1^) are the constants of the power function model. α (mg g^−1^ h^−1^) is the initial sorption rate; β (g mg^−1^)is the adsorption constant of Elovich model. k_1_ (h^−1^) and k_2_ (g mg^−1^ h^−1^) are the corresponding pseudo-first and second-order adsorption rate constans.

A chi-squared test (*X*^2^) was used to validate the adsorption kinetics models.

9 $${X}^{2}=\sum_{i=1}^{n}{\frac{({q}_{e \,exp.} - {q}_{e \,cal.})}{{q}_{e \,cal.}}}^{2}$$

where n is the number of collected data; q_*e exp*_ and q_*e cal*_ are experimentally and calculated determined quantity adsorbed at equilibrium, respectively.

## Results and discussion

### Biochar production and characterization

**Figure 1:**
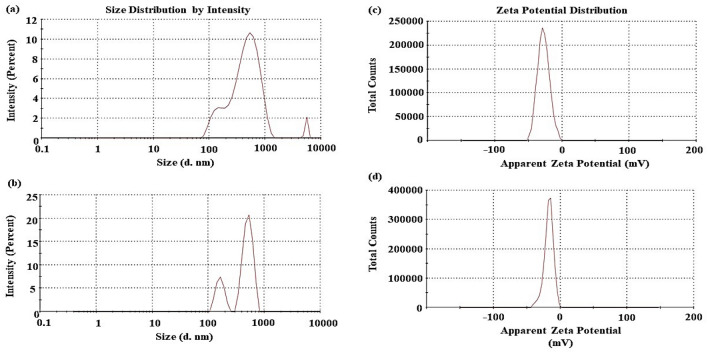
Zeta potential and Fraction curve of particle size by intensity of sunflower seed husk biochar (SHB) and (**b**) rice straw biochar (RSB) pyrolyzed at 600 °C for 30 min.: (**a**) SHB particle size distribution; (**b**); RSB particle size distribution (**c**) SHB zeta potential; and (**d**) RSB zeta potential.

The selected physicochemical properties of the sunflower seed husk (SHB) and rice straw (RSB) biochars produced at 600 °C for 30 min are shown in Table 1. The ash content in RSB is two-fold that found by SHB, which recorded 13.38% and 6.68%, respectively. Simultaneously, as ash content increased, the volatile gas percentages decreased. In other word, volatile gas percentages and ash content have an inverse trend. It means that the feedstock types had the greatest influence on the ash of produced biochars^24^. The pH values of studied biochars indicate that both materials were alkaline and relatively close with a difference value of 0.18 unit. The high pH values of the produced biochar materials could be related to concentrations of non-pyrolyzed mineral elements as well as organic matrix decomposition^15^. Additionally, the EC of both biochars were less 1 dS m^−1^. The CEC of the SHB (43.66 cmol_c_ kg^−1^) was higher than that of the RSB (34.81 cmol_c_ kg^−1^). That could be attributed to the presence of alkali and alkali metals in raw materials, which promote the formation of O-containing surface functional groups (e.g., carboxyl and phenolic groups) in the biomass with a high ash content, which eventually generate biochar with a higher CEC^24,25^. Interestingly, Ambaye et al.^1^ reported that the greater adsorption capacity would be displayed by biochar with higher CEC value. However, the higher volatile gases in HSB resulted in a higher surface area by 5.73 cm^2^ g^−1^ compared to RSB, which was 3.82 cm^2^ g^−1^, as denoted by BET analysis. Besides, the particle size distribution and zeta potential curves of both biochars are illustrated in Fig. 1. The particle size fractions of SHB (Fig. 1a) were mainly 530 nm (82.70%), 142.7 (15.0%), and small percentages had the size of 5476 nm (2.30%), while particle size fractions of RSB (Fig. 1b) were 515 nm (79.50%) and 163 nm (23.50%). In addition, as revealed by ζ-potential analysis, both biochar showed negative surface charge with a value of − 27.6 mV and − 17.3 mV for SHB and RSB, respectively (Fig. 1c and d). As indicated by Fahmi et al.^26^, ζ-potential value is positively correlated to the CEC of the biochar surface. Actually, the higher value of ζ-potential displayed by SHB reveals higher colloid stability, which implies that the lower the attraction between adjacent particles and the greater the repulsive force. That results in preventing the formation of aggregates/precipitations and guarantees the stability of dispersed particles^21^. That means the electrostatic interaction of SHB was higher than that of RSB to attract Pb^2+^ or phenol. Remarkably, the negative charge of biochar seems to be advantageous and effective in remediating cationic pollutants such as heavy metals.

**Table 1:** Basic parameters of sunflower seed husk (SHB) and rice straw (RSB) biochars produced at 600 °C for 30 min.

Parameter	Unit	SHB	RSB
Ash	%	6.68	13.38
Volatile gases	%	93.32	86.62
pH	–	10.16	9.98
EC	dS m^−1^	0.633	0.93
CEC	cmol_c_ kg^−1^	43.66	34.81
Surface area (BET)	m^2^ g^−1^	5.73	3.82
Total pore volume p/p^0^	cm^3^ g^−1^	0.04	0.02
Zeta potential	mV	− 27.6	− 17.3

**Figure 2:**
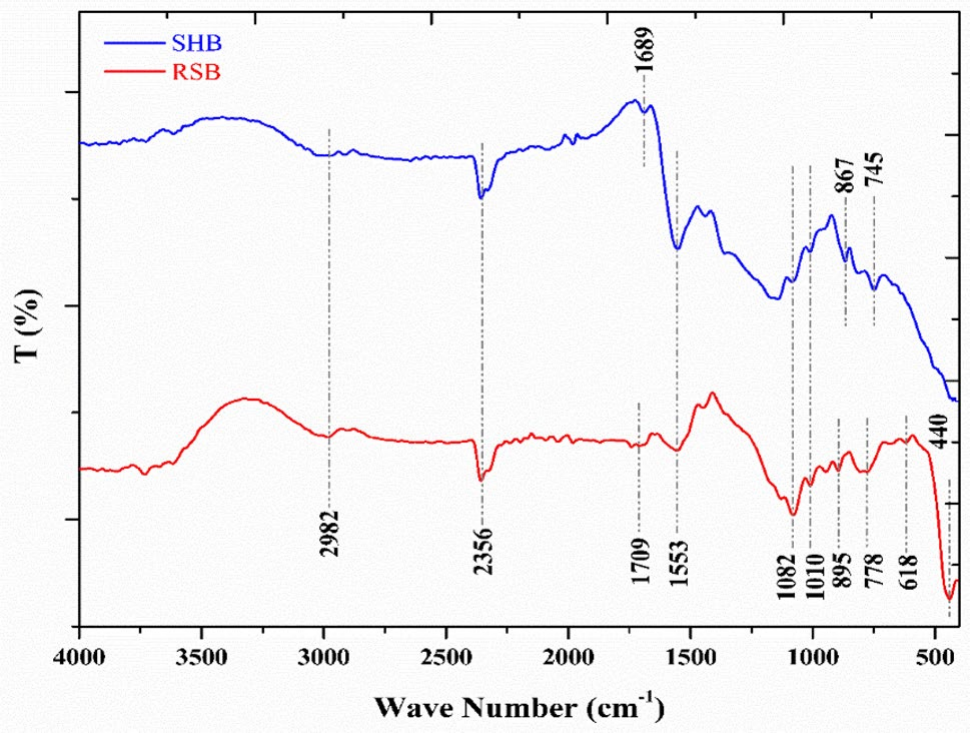
FTIR spectra of rice straw biochar (RSB) and sunflower husk seed biochar (SHB).

The main functional groups of RSB and SHB can be evidenced from the FTIR spectra ranging from 400 to 4000 cm^−1^, which depicted a noticeable differences between both biochar types (Fig. 2). FTIR pattern of both biochars indicates the presence of band at 2982 cm^−1^ corresponded to the vibration of methyl and methylene groups (Alkyl C–H Stretch). Besides, a distinguished band at 2356 cm^−1^ implied the existence of C≡N^27^. The weak band in RSB that was found at 1709 cm^−1^ is related to unconjugated carbonyl/carboxyl stretching of the existence of holocellulose and lignin^28,29^. The vibration bands at 1689 and 1553 cm^−1^ are common for aromatic compounds of C=C stretching vibration^30^. The peak at 1082 cm^−1^ indicated the presence of Si–O–Si stretching bond, while the bands at 778 and 440 cm^−1^ are related to Si–O^15,31^. Additionally, the band at 618 cm^−1^ indicates the presence of alkyl halides or may be due to the stretching variation of inorganic compounds^28^. Moreover, the spectral peaks at 618 and 895–745 cm^−1^ could be attributed to C–H, Si–H, and CH_3_ functional groups. Generally, FTIR patterns of both biochar highlightes the main functional groups that played pivotal role in Pb or phenol adsorption. These results were consistent with those obtained by El-Gamal et al.^15^; Zhao et al.^17^; Bashir et al.^30^; Tangjuank et al.^32^; Chen et al., 2011^33^.

**Figure 3:**
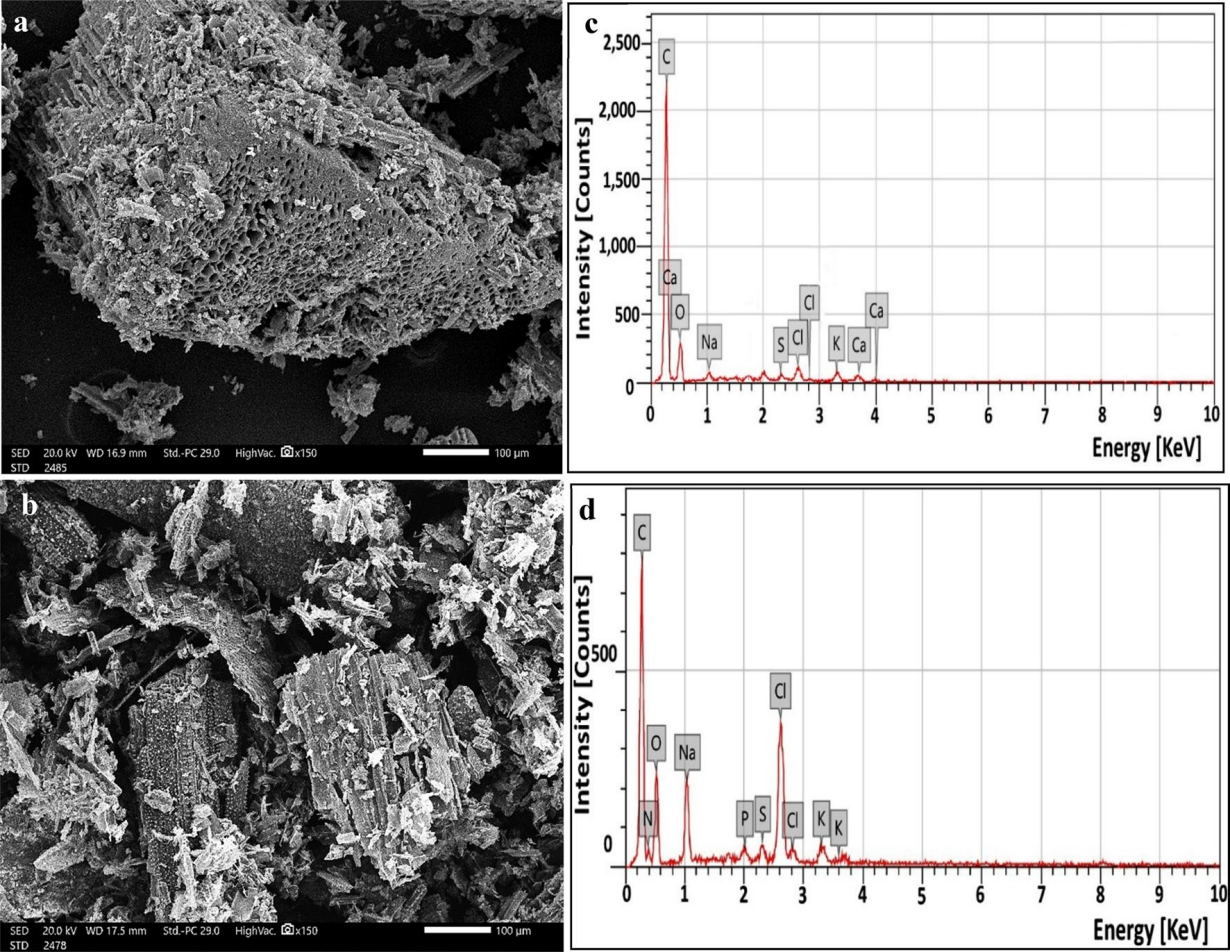
Morphological studies by scanning electron microscopy of sunflower seed husk biochar (SHB) (**a**), rice straw biochar (RSB) (**b**), and their elemental composition by EDX (**c**) and (**d**) in their respective order.

Regarding the typical morphological appearance and porous structure of parent biochars (SHB and RSB), SEM analysis was employed (Fig. 3). Despite parent SHB and RSB exhibiting coarse and rough surfaces, both biochars differed in textures and tissue structures owing to differing content of hemicellulose, cellulose, and lignin contents. Wherein, hemicellulose, cellulose, and lignin underwent dehydration reactions during the pyrolysis process, which resulted in different pore structures and sizes. As reported by Huang et al.^34^, surface structure and pore size are mainly dependent on the parent matrix of biochar, pyrolysis temperature, and degree of dehydration. Besides, Xiao et al.^35^ stated that the surface structure of biochar generated at 400 °C as pyrolysis temperature is highly adequate for immobilization purposes. However, SHB (Fig. 3a) displayed numerous channel-like structures with dissimilar diameters, which contained more pores than that RSB (Fig. 3b). Interestingly, such porous morphology is generated by volatile materials that escape during the pyrolysis process^36^. Generally, porous carriers with more surface area are highly characteristic, as they furnish considerable attachment/ adherence properties for immobilized microorganisms, are highly conducive to disseminate nutrients /metabolites and support good metabolic activity during different microbial growth phases^34,37^. In general, both studied carbonized materials with their pore features are varied, which could imply variation in their immobilizing and absorptive effectiveness.

Moreover, the chemical compositions of SHB and RSB were determined by EDX (Fig. 3c and d). As noticed, the highest signals of carbon followed by oxygen were observed indicating the presence of the primary compounds on the biochar surface. It is a plausible result as both elements symbolize the main skeleton of the agricultural waste, reflecting the presence of oxygen-containing functional groups such as carboxylic (COO −), hydroxyl (OH −), carbonyl (C = O) ester, and sulfonic groups as well as oxygen-containing inorganic phases (i.e., CO_3_^2−^, PO_4_^3−^, and SO_4_^2−^)^20^. Precisely, EDX pattern of SHB reveals that the percentages of C and O were 71.29 and 22.23, while those of RSB were 64.78 and 27.74, respectively. Besides, some other elemental fractions were also detected, such as S, P, Ca, Na, and K. It is mentioned that the fundamental structure of biochar was established by the massive bonds between organic compounds and inorganic elements^20^.

### Removal efficiency of different biochar types for lead and phenol

**Figure 4:**
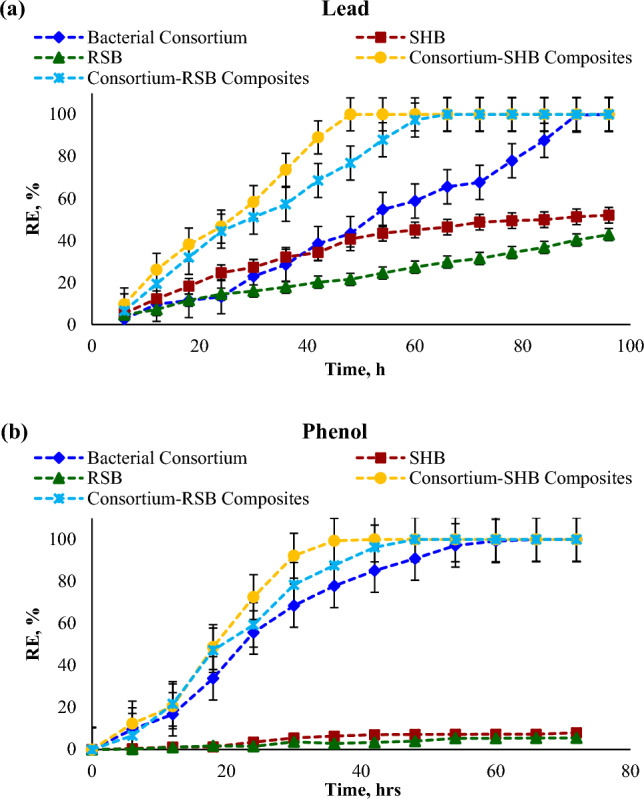
Removal efficiency (RE, %) of Lead (**a**) and phenol (**b**) through sunflower husk seed biochar (SHB), rice straw biochar (RSB), and bacterial consortium-biochar composites.

**Figure 5:**
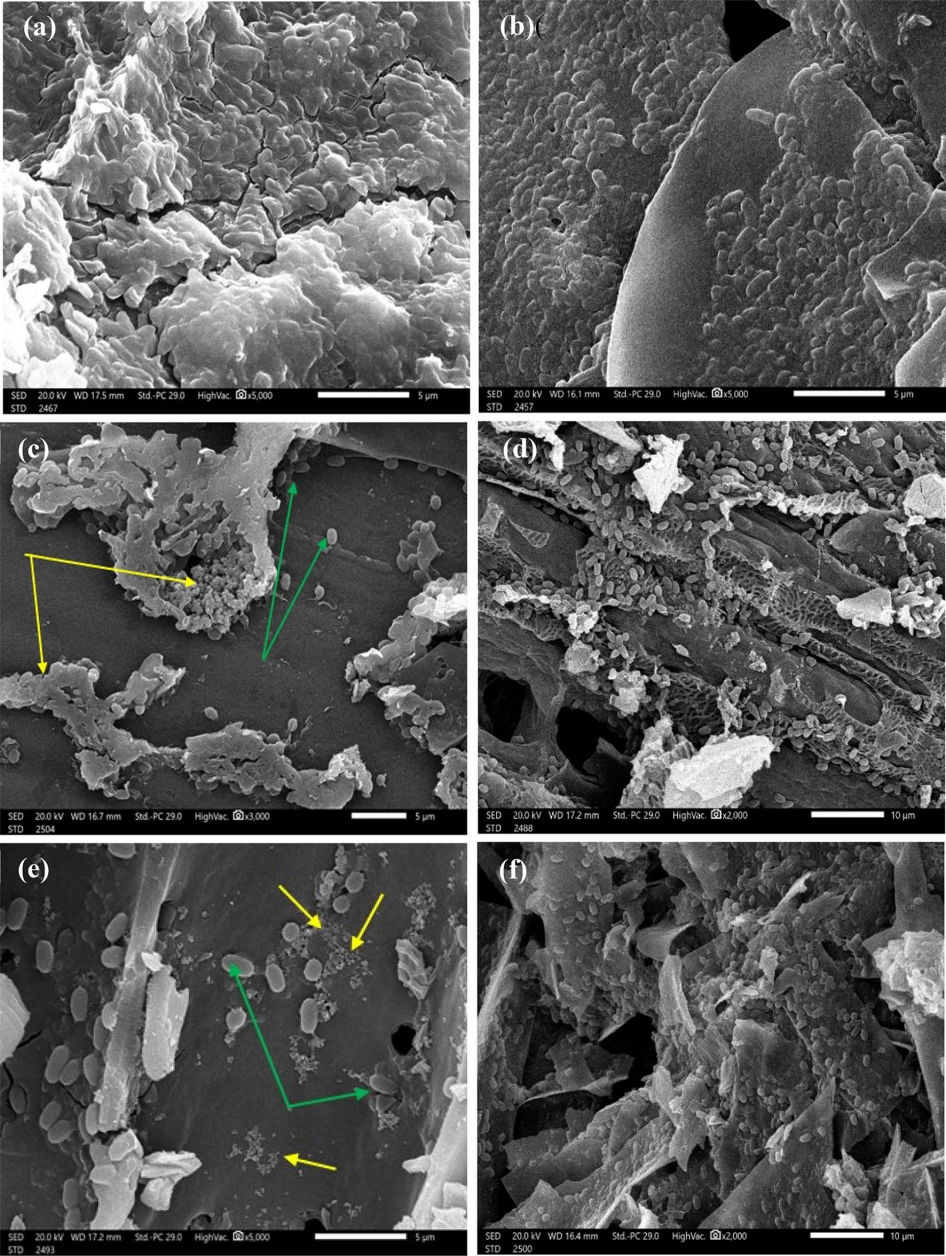
SEM micrographs of native bacterial consortium, and consortium-biochar (SHB/ RSB) composites after lead and phenol remediation (**a**) native bacterial consortium after Pb^2+^ treatment, (**b**) native bacterial consortium after phenol treatment, (**c**) Consortium-SHB composites after Pb^2+^ treatment, (**d**) Consortium-SHB composites after phenol treatment, (**e**) Consortium-RSB composites after Pb^2+^ treatment, (**f**) Consortium-RSB composites after phenol treatment, green arrows referred to bacterial cells, yellow arrows referred to Pb^2+^ precipitates.

The parent biochars of sunflower seed husk (SHB) and rice straw (RSB), besides sole bacterial consortium and consortium-biochar composites, were employed in remediating Pb^2+^ and phenol (700 mg L^−1^) from synthetic wastewater. The experiment was extended to 168 h. for Pb^2+^ and 120 h. for phenol. However, it is observed that the adsorption capacity of the bacterial consortium was achieved after 96 h. and 72 h. for Pb^2+^ and phenol, respectively (Fig. 4). Subsequently, this time was recommended to be the equilibrium time for all treatments. In general, the efficiency of the sole bacterial consortium to remove Pb^2+^ or phenol was higher than that of SHB and RSB. At the same time, the interaction of both consortium and biochar in composites improved the removal efficiency of these pollutants. Notably, the removal efficiency of parent SHB or its consortium-SHB composites exceeded that of parent RSB and its consortium-RSB composites. The bacteria’s efficiency in removing lead and phenol reached 87.64% and 90.88% after 84 h. and 48 h., respectively (Fig. 4a and b). On the other hand, the removal efficiency of Pb^2+^ by SHB and RSB reached 52.00% and 42.79% after 96 h, respectively (Fig. 4a), while it was reached to 7.99% and 5.50% for phenol during 72 h. (Fig. 4b). In comparison, complete removal of Pb^2+^ was achieved by consortium-SHB composites and consortium-RSB composites within 48 h and 66 h, respectively. In the same context, efficient remediation of phenol was implemented entirely after 42 h. and 48 h. by consortium-SHB composites and consortium-RSB composites, correspondingly. Generally, the examined consortium alone exhibited a considerable capability in eliminating 613 and 636 mg L^−1^ of Pb^2+^ and phenol, respectively, while retaining their healthy appearance and without any morphological alteration, as illustrated by SEM (Fig. 5a and b, respectively). Interestingly, bacteria represent an attractive solution for environmental biotechnologists in bioremediation processes by virtue of their resistant nature and multiple remediation strategies, which are more than displayed by eukaryotic species. Moreover, bacteria are conducted as effective biosorbent materials for toxic materials owing to their high surface-to-volume ratio and the huge number of potentially active sorption sites^12^. On the other hand, the superior adsorptive capability of SHB and its consortium-based composites could be assigned to their physicochemical traits, namely higher ζ-potential, surface area, CEC, and pH, which exceeded those displayed by RSB (Table 1). Interestingly, the alkaline pH and higher surface area of SHB with more functional groups (e.g., carboxyl, amino, hydroxyl, etc.) expedited the microbial complexation and sorption processes that ultimately improved heavy metals and organic pollutants removal from wastewater.

**Figure 6:**
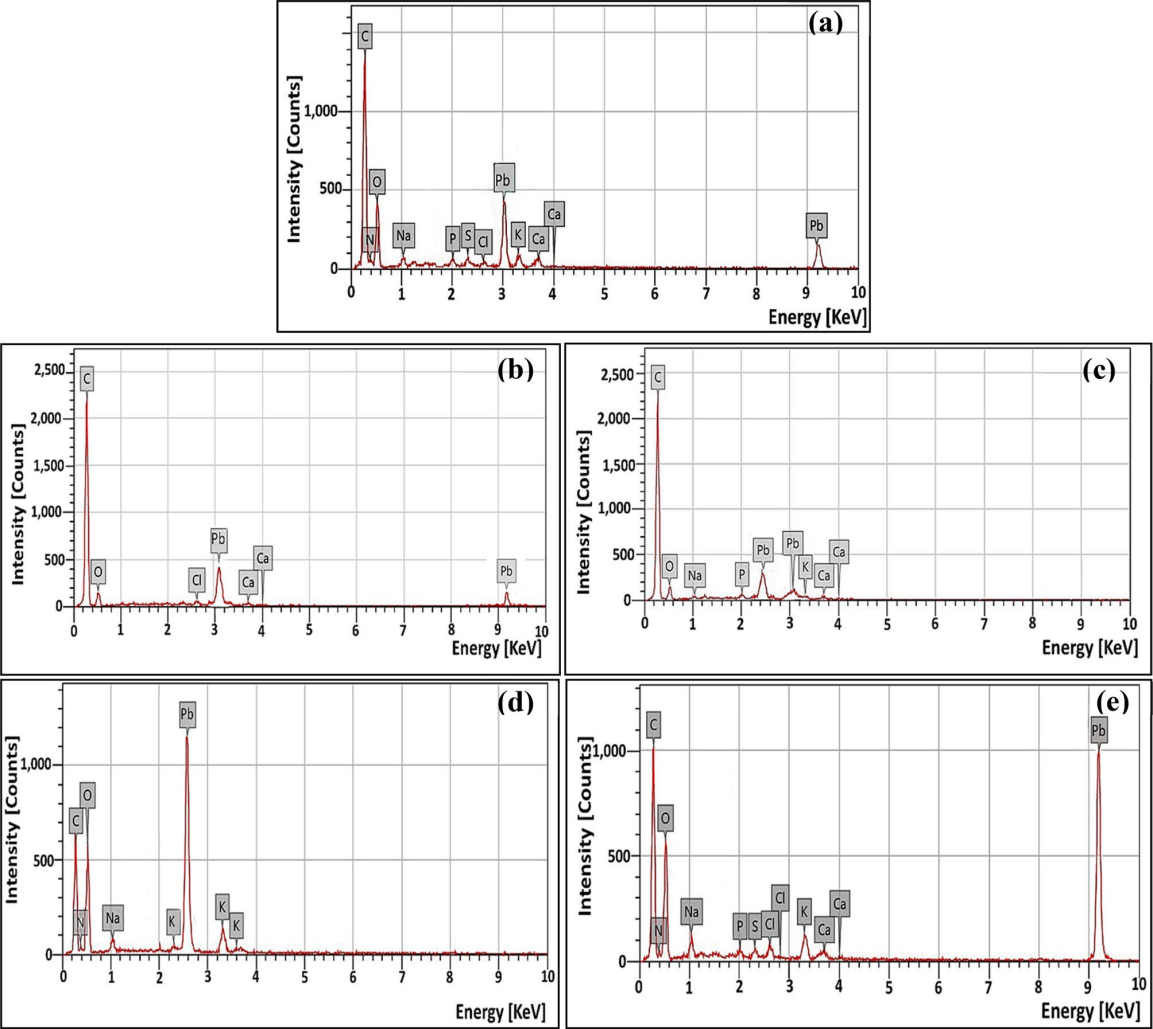
EDX patterns of native bacterial consortium, parent SHB & RSB biochar and consortium-biochar (SHB/ RSB) composites confirming lead remediation: (**a**) native bacterial consortium, (**b**) Parent SHB after Pb^2+^ treatment, (**c**) Parent RSB after Pb^2+^ treatment, (**d**) Consortium-SHB composites after Pb^2+^ treatment, (**e**) Consortium-RSB composites after Pb^2+^ treatment.

Upon hybridizing the bacterial consortium with biochar, the remediation efficacy was ameliorated. Notably, the microbial immobilization on biochar maintained its viability, sustainability, and stability and uplifted its activity. That could be ascribed to the protective role of biochar and its high surface area, which trigger the consortium in enhanced contact with the pollutant. Such claims were emphasized through SEM analysis (Fig. 5c–f), which demonstrated the compact and condensed adherence of consortium tightly on the biochar surface in a relatively uniform coverage, reflecting the fixative traits of SHB and RSB. This potent bacterial-biochar interaction could be achieved through several mechanisms such as electrostatic interaction, surface complexation, physical adsorption, cation exchange, pore filling, ion grid formation, covalent bonding, self-flocculation, polymerization, and surface precipitation^1,10,17,18^. Even after treatment and full remediation of both examined pollutants, which supposed that bacterial consortium might be exhausted from pollutant burden, the bacterial cells maintained their healthy morphology without any deformation in their shape or any changes in their textures, which unveils the adequacy and efficacy of hybridizing bacterial consortium with biochar. Via such a supportive surface (i.e., biochar), our consortium empowered full utilization of phenol as a nutrient and got rid of it entirely within the proper frame of time compared to the native consortium alone. Whereas the negatively charged cell wall of our examined consortium with carboxyl groups in lipids, in particular, enabled more binding and complexation with Pb^2+^. Thereby, the available soluble form of Pb^2+^ was removed from the solution in the form of Pb-minerals such as cerussite and anglesite, which could be formed and precipitated on the biochar surface. This suggestion was confirmed through SEM micrographs (Fig. 5c and e), which depicted bacterial cells immobilized on biochar surfaces and enclosed within precipitates. Remarkably, by the aid of the EDX examination, the elemental distribution of consortium-biochar composites after bioremediation process emphasized the complexation of Pb^2+^ on composite surfaces (Fig. 6). Notably, Chen et al.^38^ found that phosphate-dissolved bacteria-modified biochar mineralized Pb^2+^ (1000 mg L^−1^) in the formed of cerussite and pyromorphite minerals as concluded from X-ray diffraction (XRD) and Attenuated total reflection infrared spectroscopy (ATR-IR) results. However, in the mentioned study, the removal efficiency of applying phosphate solubilizing bacteria (PSB) combined with RB or SB biochar was 24.11 and 60.85%, respectively, whereas the effectiveness of PSB alone was less than 10%.

### Kinetic models

**Figure 7:**
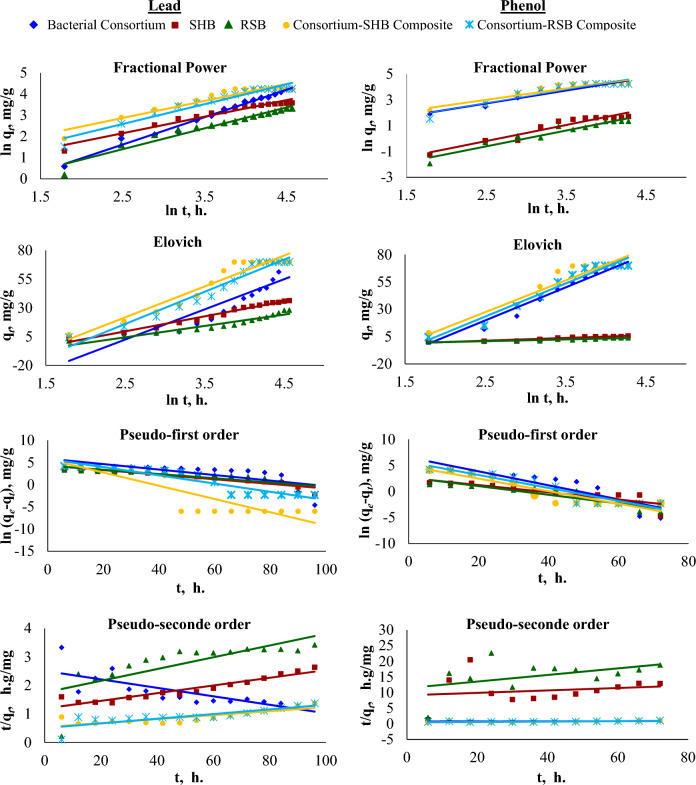
Linear correlation of kinetic models recorded for the adsorption of Pb^2+^ and phenol by bacteria, sunflower husk seed biochar (SHB), rice straw biochar (RSB), and their consortium-biochar composites.

The potential mechanism of lead and phenol adsorption onto bacterial consortium, different biochar types, and bacterial consortium-biochar composites using numerous kinetic models of Fractional power, Elovich, Pseudo-first-order (PFO), and Pseudo-second-order models (PSO) were investigated. The linear fitting kinetic parameters of the tested models are presented in Tables 2 and 3, the while kinetic curves of Pb^2+^ and phenol are displayed in Fig. 7. The Fractional power model is the modified form of the Freundlich equation, while Elovich model has been extensively used in the explication of chemisorption processes that follow second-order kinetics of the heterogeneous surfaces. PFO assumes that the number of adsorbate molecules could adsorb onto the same number of biosorbent active site, as well as PSO depends on the sharing and exchanging of electron between pollutants and adsorbent that meaning the adsorption is chemical process^39,40^.

**Table 2:** Kinetic parameters of fitting kinetic models for the lead adsorption onto native bacterial consortium, parent sunflower seed husk biochar (SHB), rice straw biochar (RSB), and consortium-biochar (SHB/ RSB) composites.

Treat	Bacteria	SHB	RSB	Consortium-SHB Composites	Consortium-SHB Composites
q_*e,exp*_ (mg g^−1^)	70.00	36.50	28.18	70.00	70.00
Fractional power					
a (mg g^−1^)	0.20	1.21	0.34	2.35	1.27
b (h^−1^)	1.30	0.78	0.99	0.81	0.94
R^2^	0.993	0.981	0.973	0.954	0.972
RMSE	0.123	0.127	0.192	0.206	0.187
* X*^*2*^	0.015	0.016	0.037	0.043	0.035
Elovich					
α (mg g^−1^ h^−1^)	0.003	0.013	0.013	0.007	0.005
β (g mg^−1^)	26.27	13.08	9.88	26.88	28.02
R^2^	0.910	0.989	0.959	0.963	0.977
RMSE	9.783	1.570	2.374	6.127	4.947
* X*^*2*^	95.71	2.47	5.63	37.54	24.47
PFO					
K_1_ (h^−1^)	− 0.06	− 0.05	− 0.05	− 0.15	− 0.09
q_*e,cal*_ (mg g^−1^)	361.13	75.28	73.86	309.4	338.6
R^2^	0.733	0.938	0.835	0.883	0.933
RMSE	1.700	0.562	0.928	2.353	1.059
* X*^*2*^	2.89	0.32	0.86	5.54	1.12
PSO					
K_2_ (g mg^−1^ h^−1^)	7.66*10^−6^	17.30*10^−6^	19.39*10^−6^	11.55*10^−6^	14.48*10^−6^
q_*e,cal*_ (mg g^−1^)	− 72.622	70.373	53.362	129.534	117.925
R^2^	0.788	0.963	0.760	0.879	0.868
RMSE	0.33	0.12	0.49	0.13	0.15
* X*^*2*^	0.11	0.02	0.24	0.02	0.02

*q_*e,exp*_, Experimental adsorption capacities; q_*e,cal*_, Calculated adsorption capacities.

PFO, Pseudo-First order; PSO, Pseudo-Second order.

R^2^, Regresion coeffient; RMSE, Root mean square error; *X*^2^, Chi-square.

**Table 3:** Kinetic parameters of fitting kinetic models for phenol adsorption onto native bacterial consortium, parent sunflower seed husk biochar (SHB), rice straw biochar (RSB), and consortium-biochar (SHB/ RSB) composites.

Treat	Bacteria	SHB	RSB	Consortium-SHB composites	Consortium-SHB composites
q_*e,exp*_ (mg g^−1^)	69.99	5.59	3.83	69.90	69.90
Fractional power					
a (mg g^−1^)	1.25	0.04	0.03	2.25	1.16
b (h^−1^)	1.00	1.24	1.23	0.88	1.04
R^2^	0.973	0.959	0.963	0.932	0.944
RMSE	0.19	0.29	0.27	0.27	0.29
* X*^*2*^	0.04	0.08	0.07	0.07	0.08
Elovich					
α (mg g^−1^ h^−1^)	0.01	0.06	0.08	0.01	0.01
β (g mg^−1^)	30.12	2.48	1.64	28.94	30.20
R^2^	0.980	0.954	0.943	0.946	0.977
RMSE	4.82	0.62	0.46	7.89	5.19
* X*^*2*^	23.20	0.39	0.21	62.27	26.90
PFO					
K_1_ (h^−1^)	− 0.13	− 0.07	− 0.08	− 0.12	− 0.12
q_*e*,*cal*_ (mg g^−1^)	697.50	13.60	14.88	130.26	284.22
R^2^	0.897	0.864	0.912	0.920	0.944
RMSE	0.79	0.72	0.82	0.83	0.88
* X*^*2*^	2.26	0.84	0.73	1.35	0.97
PSO					
K_2_ (g mg^−1^ h^−1^)	0.36*10^−5^	16.85*10^−5^	97.16*10^−5^	13.52*10^−5^	6.59*10^−5^
q_*e*,*cal*_ (mg g^−1^)	609.76	25.54	9.50	137.36	175.75
R^2^	0.24	0.19	0.45	0.75	0.75
RMSE	0.15	4.69	4.69	0.14	0.11
* X*^*2*^	0.02	22.04	21.97	0.02	0.01

* q_*e,exp*_, Experimental adsorption capacities; q_*e,cal*_, calculated adsorption capacities.

PFO, Pseudo-First order; PSO, Pseudo-Second order.

R^2^, Regresion coeffient; RMSE, Root mean square error; *X*^2^, Chi-square.

According to correlation coefficients (R^2^) values of the studied models, Elovich model is the best model to describe that Pb^2+^ and phenol adsorption due to the highest correlation coefficients that ranged from 0.91 to 0.99 for Pb^2+^ and 0.95 to 0.98 for phenol, closely followed by the Fractional Power for Pb^2+^ (0.95 and 0.99, Table 2) and phenol (0.93 to 0.97, Table 3). The R^2^ values of the PFO model were lower than those of the two mentioned models, with values of 0.73 for Pb^2+^ by the bacterial consortium treatment (Table 2) and 0.86 for phenol by the SHB treatment (Table 3). However, the PSO kinetic model does not show a decent correlation for the adsorption of Pb^2+^. On the other hand, phenol's PFO correlation coefficient is markedly higher than PSO. Remarkably, Pseudo-second-order model is ineffective in describing the adsorption process of phenol by all biochar types due to the lower calculated R^2^ ranged from 0.19 to 0.75 (Table 3). Phenol adsorption was best described by the Fractional power and Elovich models as indicated by the highest correlation coefficients. Additionally, the calculated adsorption capacities (q_*e,cal*_) values were significantly different than the values of experimental adsorption capacities data (q_*e,exp*_). Generally, the q_*e,cal*_ values of Pb^2+^ and phenol were extremely high for bacterial consortium and consortium-biochar composites, while for both biochar types were relatively high. Consequently, the high concentration of Pb^2+^ and phenol adsorbed by different biomaterial did not follow Pseudo-first-order and Pseudo-second-order equations. In contrast, the adsorption process of phenol (50 mg L^−1^) by three pine fruit shells (10 g L^−1^) at 25 and pH 6.5 confirmed that the pseudo-second order kinetic model was the best one depending correlation confident (R^2^ > 0.999) followed by Elovich kinetic models^41^. In addition, numerous studies confirmed that the PFO model could not represent adsorption of heavy metals by various biochar types^19,42,43^.

However, in this study, the initial rate sorption of Elovich (α, mg g^−1^ h^−1^) was the highest by both biochar types, either for Pb^2+^ (0.013 mg g^−1^ h^−1^) or phenol (SHB, 0.06 mg g^−1^ h^−1^ and RSB, 0.08 mg g^−1^ h^−1^) rather than bacterial consortium and consortium-biochar composites. Subsequently, the order of this value could be summarized as SHB = RSB > consortium-SHB composites > consortium-RHB composites > Bacteria for Pb^2+^ and RSB > SHB > consortium-SHB composites ≥ consortium-RHB composites ≥ Bacteria for phenol. Depending on Elovich model parameter, higher values of α for SHB and RSB illustrated a better adsorption mechanism and suggests that chemisorption is the rate-limiting^39,44^. Furthermore, the low value of the Elovich constant β indicates that chemisorption activation energy is lower and the adsorption process occurs quickly*,* which was fairly low for both biochars to remove Pb^2+^ and phenol relative to the bacterial consortium and consortium-biochar composites. Inyinbor et al.^44^ concluded that increasing α value with increasing initial concentration, while β value decreased indicating that more than one mechanism governs the adsorption process. β is a constant associated with the extension of surface coverage, the surface area of biochar was found to be relatively low, implying that biochar adsorption may occur more through functional groups. In the study to remove of Sr^2+^ (50 and 150 mg L^−1^) using sugarcane bagasse biochar through Stirred-batch technique at different time, Salem (2023)^43^ reported that the higher the concentration, the higher α value and lower β value. This proposed that the reaction between the biochar and adsorbant is a chemical reaction^44^. According to the Fractional power model, the adsorption rate (a, mg g^−1^) of Pb^2+^ and phenol by consortium-SHB composites recorded the highest value by 2.35 mg g^−1^ and 2.25 mg g^−1^ followed by consortium-RHB composites for Pb^2+^ and bacterial consortium for phenol, which were 1.27 mg g^−1^ and 1.25 mg g^−1^, respectively. While bacterial consortium and both biochar types recorded the lowest value for lead and phenol, respectively. Additionally, the b value of Fractional Power constant was lower than unity (b < 1) in both biochar types and consortium–biochar composites for lead and bacterial consortium only and consortium–biochar composites for phenol meaning that this model may describe the adsorption kinetics data well^44^.

It reported that the highest correlation coefficient (R^2^) value and the lower residual root mean square error (RMSE) and chi-square (*X*^*2*^) values are the best indication to fit the adsorption model and describe the adsorption process^39^. However, Syafiuddin et al.^45^ reported that the results of ranking fifteen kinetic models for modeling the adsorption of silver nanoparticles do fit to the experimental data that depending on the type of adsorbent.

## Conclusion

To summarize, the feasibility of *Proteus mirabilis* (10B) and *Raoultella planticola* (VIP) strains immobilized on seed husk (SHB) and rice straw (RSB) biochar in remediating 700 mg L^−1^ of Pb^2+^ and phenol was evaluated. The pyrolyzed SHB and RSB were characterized using SEM, FTIR, BET and ζ-potential. Besides, other physicochemical parameters also were determined (e.g., ash content, pH values, EC and CEC). Both SHB and RSB were used as matrix to immobilize the bacterial consortium, which was applied subsequently in the bioremediation approach. The current study showed that removal effeciency of both pollutants was maximized in shorter time after employing consortium-biochar composites, relative to individual bacterial consortium or parent biochars. Wherein, consortium-biochar composites remediated Pb^2+^ entirely after 48 h and 66 h by consortium-SHB composites and consortium-RSB composites, respectively. On the other hand, complete elimination of phenol was detected within 42 h and 48 h by both consortium-biochar composites in their respective order. SEM and EDX of the consortium-biochar composites illustrated a better understanding of the Pb^2+^ and phenol biosorption mechanism. In addition, the Elovich kinetic and Fractional Power models was the best model to describe adsorption capacity of high concentration of Pb^2+^ and phenol rather than Pseudo-first-order, and Pseudo-second-order. Generally, this study represents a complying paradigm for the concept of remediating waste with waste by hybridizing waste biomass-based biochar, as a carrier adsorbent, with promising remediating bacterial consortium. As a primary study to evaluate the proposed biosorbent materials, this study provides valuable information relative to efficiency of the mixture of bacteria and biochar to remove inorganic and organic pollutants from wastewater. Finally, this study needs further applications to evaluate the performance of proposed consortium-biochar composites as biosorbent materials to eliminate a wide range of contaminates of real wastewater, which we will focus on in our next study.

## Data Availability

All data generated or analyzed during this study are included in this article.
